# Local oceanographic variability influences the performance of juvenile abalone under climate change

**DOI:** 10.1038/s41598-018-23746-z

**Published:** 2018-04-03

**Authors:** C. A. Boch, F. Micheli, M. AlNajjar, S. G. Monismith, J. M. Beers, J. C. Bonilla, A. M. Espinoza, L. Vazquez-Vera, C. B. Woodson

**Affiliations:** 1 Hopkins Marine Station, Stanford University, Pacific Grove, CA 93950 USA; 2 Monterey Bay Aquarium Research Institute, Moss Landing, CA 95039 USA; 3 Department of Civil and Environmental Engineering, Stanford University, Stanford, CA 94305 USA; 4 Sociedad Cooperativa de Producción Pesquera La Purisima, Bahia Tortugas, Baja California Sur Mexico; 5 Sociedad Cooperativa de Producción Pesquera Buzos y Pescadores, Isla Natividad, Baja California Sur Mexico; 6 Comunidad y Biodiversidad A.C., Calle Isla del Peruano No.215, Guaymas, Sonora 85448 Mexico; 7 College of Engineering, University of Georgia, Athens, GA 30602 USA

## Abstract

Climate change is causing warming, deoxygenation, and acidification of the global ocean. However, manifestation of climate change may vary at local scales due to oceanographic conditions. Variation in stressors, such as high temperature and low oxygen, at local scales may lead to variable biological responses and spatial refuges from climate impacts. We conducted outplant experiments at two locations separated by ~2.5 km and two sites at each location separated by ~200 m in the nearshore of Isla Natividad, Mexico to assess how local ocean conditions (warming and hypoxia) may affect juvenile abalone performance. Here, we show that abalone growth and mortality mapped to variability in stress exposure across sites and locations. These insights indicate that management decisions aimed at maintaining and recovering valuable marine species in the face of climate change need to be informed by local variability in environmental conditions.

## Introduction

Climate-driven collapses of coastal marine populations pose major threats to marine ecosystems and the services they provide worldwide^1,2^. Many coastal fisheries have recently collapsed from climate driven stressors with significant economic losses and alteration of ecosystem function^1,3,4^. Following collapse, species, fisheries, and ecosystems often show no signs of recovery^2,5,6^. Yet, some populations appear to be resistant to these events, or able to rapidly recover^7–9^. A key factor influencing the extent of climatic impact and the resilience of affected populations is the spatial scale and heterogeneity of extreme events. Mass mortalities can be widespread resulting in a decline of affected species with slow recovery^10,11^. However, mortality has also been reported to be sometimes patchy, resulting from local adaptation or spatial refuges allowing for quicker recovery^12,13^. Thus, while documented evidence of these mortality events continue to mount, understanding and predicting the key oceanographic factors that allow for recruitment survival and adaptation in nearshore ecosystems remains lacking and thus require significant attention.

Anomalous high temperatures or temperature-induced disease outbreaks are generally considered the major causes of abalone mass mortality^14–16^ despite positive effects of warm temperatures on growth rates of abalone^17,18^. In contrast to temperature, low dissolved oxygen has been shown to have negative effects on growth and survival of abalone^19,20^. However, despite the understanding that temperature and dissolved oxygen play major roles in both growth and mortality, the contribution of temperature stress as it co-varies with low oxygen remains unclear. For example, low levels of dissolved oxygen can sequentially occur with high temperatures in nearshore systems due to eutrophication, changes in EÑSO, mixed layer dynamics, and wind forcing^21–25^. Such dynamics can vary widely over relatively small distances (100–1000 s of m). Despite the global predictions for warming oceans and increased low dissolved oxygen waters in nearshore systems, understanding how large-scale phenomena manifest and affect biological processes at the local spatial scales remains largely limited to inference from laboratory investigations without consideration of local oceanographic variability.

**Figure 1:**
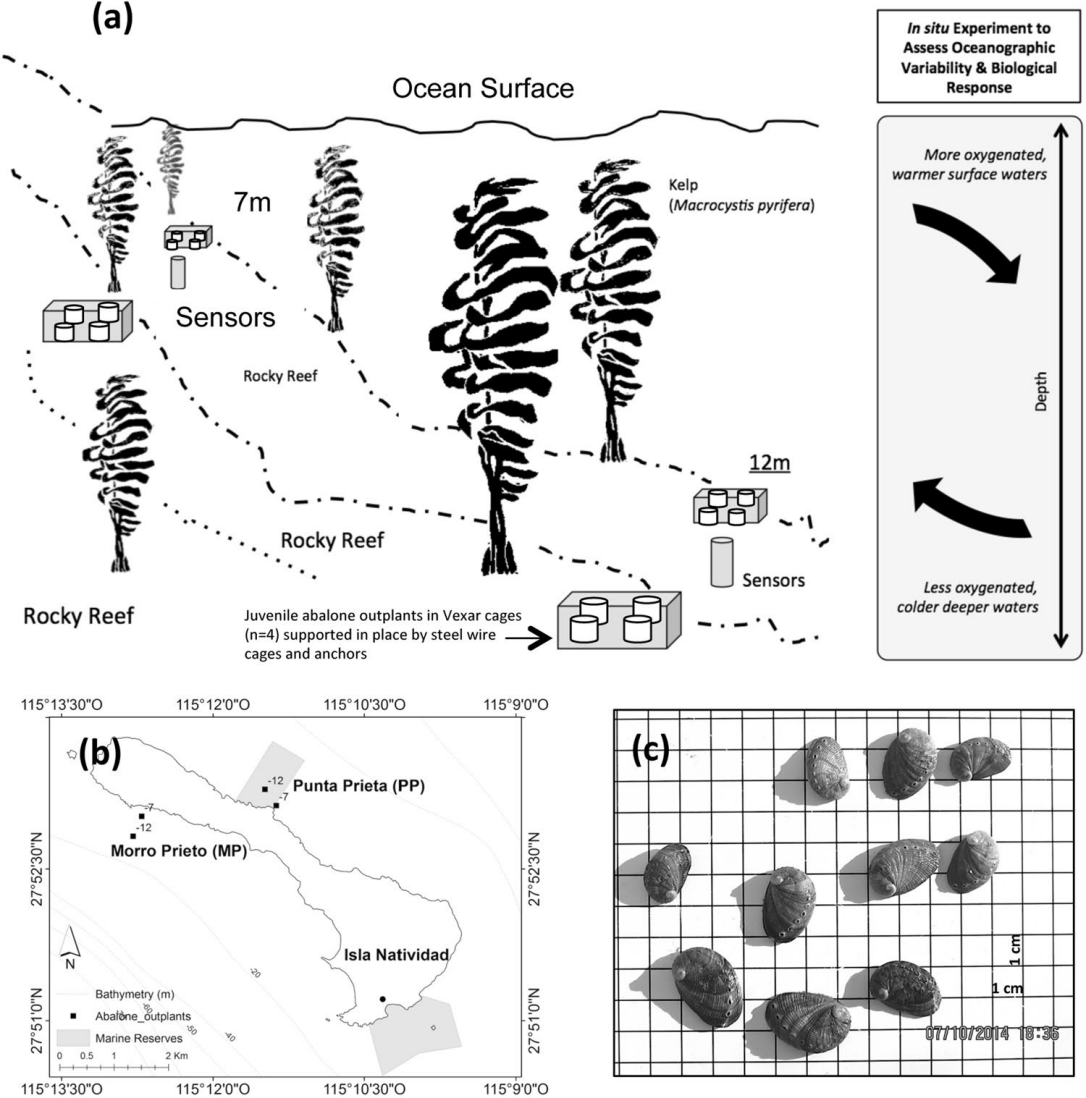
Field experiments assessing juvenile abalone performance under local oceanographic variation. **(a)** Field experimental design. Juvenile abalone were placed in experimental Vexar cages placed within large steel wire mesh cages at different depths and locations to quantify the effects of seawater surface warming and upwelling dynamics on abalone growth and mortality. **(b)** Map of the experimental site (Isla Natividad, Baja California, Mexico). Light-grey polygons are designated marine reserves. Black solid squares mark the 4 experimental sites—2 at Morro Prieto on the West (Pacific Ocean side) and 2 at Punta Prieta Reserve on the East (Bahia Vizcaino side). Island map and bathymetry (Source: INEGI http://www.beta.inegi.org.mx/app/biblioteca/ficha.html?upc=889463039839 (2010) and http://www.inegi.org.mx/geo/contenidos/datosrelieve/submarino/batrimetrica_zeesv.aspx (2017) respectively) was modified in ArcGIS (v10.2.2) to delineate reserve polygons and site locations. **(c)** Juvenile abalone from aquaculture facilities were placed on a 16 cm × 16 cm grid (1 cm^2^ increments) and photographed for standardized shell length measurements 1 day prior to deployment and on the final day of experiments.

In the shallow water kelp forest reefs of Isla Natividad (Fig. 1a,b; 27 N, 115 W; Baja California, Mexico), adult green abalone (*Haliotis fulgens)* are distributed on both sides of the island from the intertidal to ~18 m in depth. Monitoring of abalone populations has been conducted at this location since 2006, and mass mortalities were documented in 2009–2010^26^. However, information that would help predict recruitment patterns is lacking especially under an accelerating changing climate. Coastal oceanographic monitoring using nearshore moorings indicate that Morro Prieto, which is on the Pacific side of the island, is generally colder and more exposed to upwelling events. In contrast, Punta Prieta, which is on the Vizcaino Bay side of the island, is generally warmer and less exposed to upwelling. Using the two sides of the island as a natural laboratory during the upwelling season, we examined how large-scale forcing manifested at depth and in horizontal space, in terms of temperature and dissolved oxygen exposure. Within this exposure to covariates, we examined the growth and mortality response of juvenile abalone outplants—coupled with laboratory temperature ramp experiments—to gain insights into *in situ* biological responses to oceanographic variability.

We hypothesized that exposure to low dissolved oxygen levels that are typically associated with upwelling events would have negative impacts on growth and mortality. However, it is difficult to make predictions regarding the effects of low dissolved oxygen combined with higher temperatures on growth and mortality due to the limited knowledge of the effects of these covariates. We hypothesized that the combined exposure to low dissolved oxygen and high temperatures was likely to have a negative influence on growth and survival because high temperatures can increase metabolic rates and oxygen demand, thereby possibly exacerbating the impacts of low oxygen conditions.

## Methods

### Oceanographic Conditions

**Figure 2:**
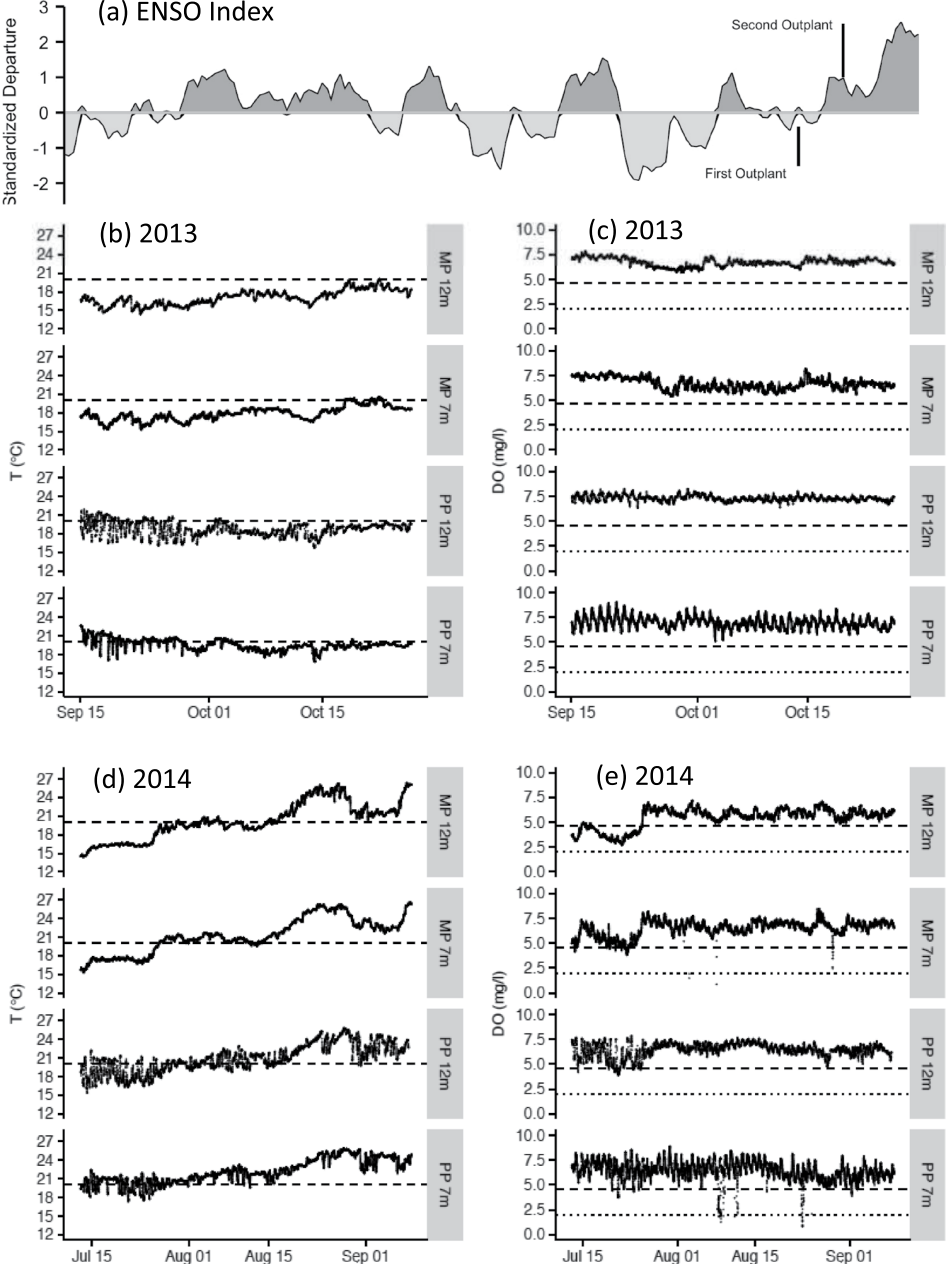
Climate and local oceanographic conditions during the *in situ* abalone experiments. **(a)** Recent NOAA estimates of ENSO oscillations (from https://www.esrl.noaa.gov/psd/enso/mei/table.html). Initial times for both abalone outplants are indicated on the index. (**b**,**d**) Sep. – Oct. 2013 and July – Sep 2014 time series of seawater temperature (°C) at the 4 abalone outplant locations. Dashed black lines represent the 20 °C temperature reference used to derive the Temperature Index (TI). **(c**,**e)** Sep – Oct 2013 and July – Sep 2014 time series of dissolved oxygen concentration (mg/l) at the 4 outplant locations. Long dashed black lines represent the sub-lethal levels of dissolved oxygen (4.6 mg/l) used to derive the Oxygen Index (OI). Short black dashed lines represent the approximate lethal levels of dissolved oxygen (2.0 mg/l) reported in literature for many marine invertebrates (Vaquer-Sunyer and Duarte, 2008). For panels (**b**–**e**), labels on the right side of the panels indicate the location and depth of the sensors. MP = Morro Prieto; PP = Punta Prieta.

In order to understand the nearshore oceanographic variability in our study region under the context of regional climate variability, we used the multivariate EÑSO Index (MEI)^27^ to examine any climatic anomalies the abalone may have experienced during the experiments (Fig. 2a). We also obtained wind data from the NOAA/NCDC Blended Daily 0.25-degree sea surface wind data set that is available on the NOAA-ERDDAP data portal for the period from 2002–2016. We used satellite-derived winds to estimate local upwelling between years in terms of cumulative wind stress^28^. We first rotated the winds into along- and across-shore components. We then computed the cumulative wind stress by integrating the wind stress record by year and normalizing to the beginning of the upwelling period^28,29^.

To characterize differences in ocean conditions at each site (MP and PP) and depth (7 m or 12 m) (Fig. 1), dissolved oxygen and temperature were measured continuously during experiments, in 2013 and 2014, using either a Seabird SBE37-ODO (Sea-Bird Electronics, Inc., Washington, USA), an Aanderaa Optode (Xylem Inc., New York, USA) or a PME MiniDOT (Precision Measurement Engineering, Inc., California, USA) sensor. Each sensor was cross-calibrated with other sensors prior to and after deployment, and sampled at 10 minute intervals through the duration of the experiment. Data were downloaded at the end of 9 weeks for both years and variation in these variables were compared between sites, depths and through time to characterize the environmental conditions to which each group of animals were exposed (e.g. the occurrence, intensity and duration of hypoxic events, see Fig. 2).

### Reduction of oceanographic variables

We used the temperature and oxygen time series to calculate several exposure parameters including the number of extreme events, event intensity, rate of change, and integrated exposure. Reference points were established through examination of historical time series and biological relevance identified in the literature. We used 20 °C as a relevant threshold for temperature because bottom water temperatures at Isla Natividad rarely exceed this value, except during positive EÑSO years. For dissolved oxygen (DO), we used 4.6 mg/l as a relevant threshold because this level has been reported to be physiologically stressful for many marine invertebrates^22^.

An extreme event was defined as a period greater than 2 hours where T > 20 °C or DO < 4.6 mg/l. We identified events by subtracting the reference from the raw data, then setting all values below 20 °C or above 4.6 mg/l to zero. We then calculated several parameters from these modified time series. Intensity of an event was defined as the absolute value above (maximum ΔT) or below (minimum ΔDO) the reference point during the event. Duration of exposure was the length of time above or below the reference point for each event. Rate of change was defined as the intensity of an event divided by the time for an event to reach peak intensity. Integrated exposure was defined as the integral over the time of the stressor above or below the reference point between each ecological sampling period.

Ten predictor variables were estimated and reduced from measurements of both temperature and DO: mean and coefficient of variation for both temperature and DO, number of extreme temperature and DO events, maximum temperature, minimum dissolved oxygen, integrated exposure to high temperature, and integrated exposure to low oxygen. All variables were calculated for each week of the experiments, to match the temporal scale at which juvenile mortality was quantified. We then used principle component and regression analysis to determine which variables were significantly correlated and contributed the most variance from week to week during each experiment. We found that the mean T, maximum T, and integrated T exposure were all highly correlated and grouped into PC1—with the highest loading for integrated T exposure. Furthermore, we found that integrated T exposure exhibited the greatest variability between the sites and depths. Similar results occurred for DO. Integrated DO exposure was the variable exhibiting the most variation during the experiments. This approach allowed us to reduce the ten original variables to four uncorrelated parameters: integrated T and DO exposure, and the number of T and DO extreme events.

Using the four parameters identified in the reduction of the oceanographic data, we further reduced the covariates of temperature and dissolved oxygen to Temperature (TI) and Oxygen Indices (OI) to analyze the effects of these variables as a total exposure on the growth and mortality of juvenile abalone. TI and OI were computed as the integrated exposure divided by the number of events. In this sense, higher index values indicate longer exposure to longer individual events whereas lower index values may indicate extensive exposure over frequent events or little exposure.

### Growth and mortality response to seasonal upwelling drivers

The first field experiment was conducted between late August and late October 2013 to test the growth and mortality responses to possible high temperatures during upwelling relaxation, and upwelling-related hypoxic events. Juvenile green abalone, *Haliotis fulgens* (1.44 ± 0.12 cm, mean length ± s.d.), were deployed at 7 and 12 m in depth (Figs S1 and S2) on two sides of Isla Natividad (Fig. 1a,b). These depths were targeted because green abalone range from the intertidal to ~12 m (and as deep as 18 m at some locations), with the greatest abundances between 5–8 m. Thus, the shallow deployments (7 m) correspond with the depth where this species is most abundant, and the deeper deployments (12 m) are at the edge of the depth distribution for green abalone, where animals may experience the most stressful conditions, particularly periodic exposure to hypoxic waters. We placed n = 12 abalone in four Vexar mesh cages (Fig. S1) to keep the groups of abalones separate from each other and facilitate weekly assessment of their survivorship. Each compartment contained a sheet of fiberglass to provide a hard surface for the animals to attach. These compartments were placed within a larger steel wire metal cage (85 × 70 × 30 cm; Fig. S2) that provided structure and support. In total, n = 384 juvenile abalone in 32 Vexar cages, were outplanted in a total of 8 steel wire cages for this experiment, with 2 replicate steel cages placed ~10 m apart at each of four field sites: MP7m, MP12m, PP7m and PP12m (Fig. 1). All juvenile green abalones for the 2013 experiment were cultured in the Isla Natividad aquaculture facility from 11 adults collected at Morro Prieto.

The field outplant experiment was repeated in July to early September of 2014, to target upwelling and upwelling relaxation periods. Juvenile abalone (1.99 ± 0.08 cm, mean length ± s.d.) were tested using the same methods described for 2013—i.e., through monitoring the performance of outplanted juveniles and of oceanographic conditions at different depths and locations. A second cohort of juvenile abalone was acquired from Isla Natividad abalone aquaculture (~16 months old, n = 320 total, n = 20 per compartment) and were again placed in Vexar mesh compartments within steel wire cages (n = 2 Vexar cages/steel wire cage, 2 replicate steel cages at each site and depth combination) at the same Morro Prieto and Punta Prieta locations and depths of 2013 (Fig. 1a). An additional set of juvenile abalone (n = 40) were cultured in the laboratory in Vexar cages (n = 2 cages, 20 individuals/cage) placed in flow through tanks as a comparative reference to the outplants. A MiniDOT dissolved oxygen and temperature sensor was placed next to the cages to record incoming seawater conditions throughout the experiment.

To quantify the mean changes in shell size as an indicator of growth, we took photographs of each replicate abalone group 1 day prior to deployment (Size_Initial_) and on the final day of the experiment (Size_Final_). To minimize handling stress and processing time, replicate groups of animals were placed on a waterproof paper with a 16 cm × 16 cm grid, dabbed dried with tissue paper to remove water distortion, photographed with a Canon S10 Powershot camera (Canon USA Inc.) mounted on a tripod, and immediately placed back in flow-through tanks. We post-processed each of the images using ImageJ software (https://imagej.nih.gov/ij/). To minimize camera lens distortion of the image, 1 cm section of the reference grid immediately next to each juvenile abalone was used to set the image scale. Once the scale was defined in ImageJ, abalone shell diameter was then estimated using the ruler tool. To estimate individual growth, Size_Initial_ was subtracted from Size_Final_ after visually confirming the unique color and banding patterns on the surface of the shell at the two different time points (Fig. S1D,E).

In order to quantify weekly mortality and survivorship, SCUBA divers visited each cage for both the 2013 and 2014 experiments as weather and oceanic conditions permitted. During each visit, empty shells were collected from the compartments and placed into pre-labeled Ziploc bags—i.e, labeled with cage and compartment number. Surviving abalone were fed giant kelp (*Macrocystis pyrifera*) *ad libitum* during each visit. Kelp was provided in excess and was never completely consumed over the week intervals, indicating food was not limiting through the experiments. We defined mortality as the number of empty shells collected from each Vexar cage and the remaining abalone as alive. For subsequent weekly censuses, we subtracted any new number of empty shells from the remaining number of abalone alive from the previous week. Thus, the proportion of dead abalone relative to the total abalone for each week was iteratively adjusted after counting any empty shells. Dead individuals were not replaced with live ones at the weekly inspections because our objective was to follow the fate of groups of animals exposed to the same conditions, eliminating the confounding of different histories.

### Behavioral, physiological and demographic effects of high temperature

**Figure 3:**
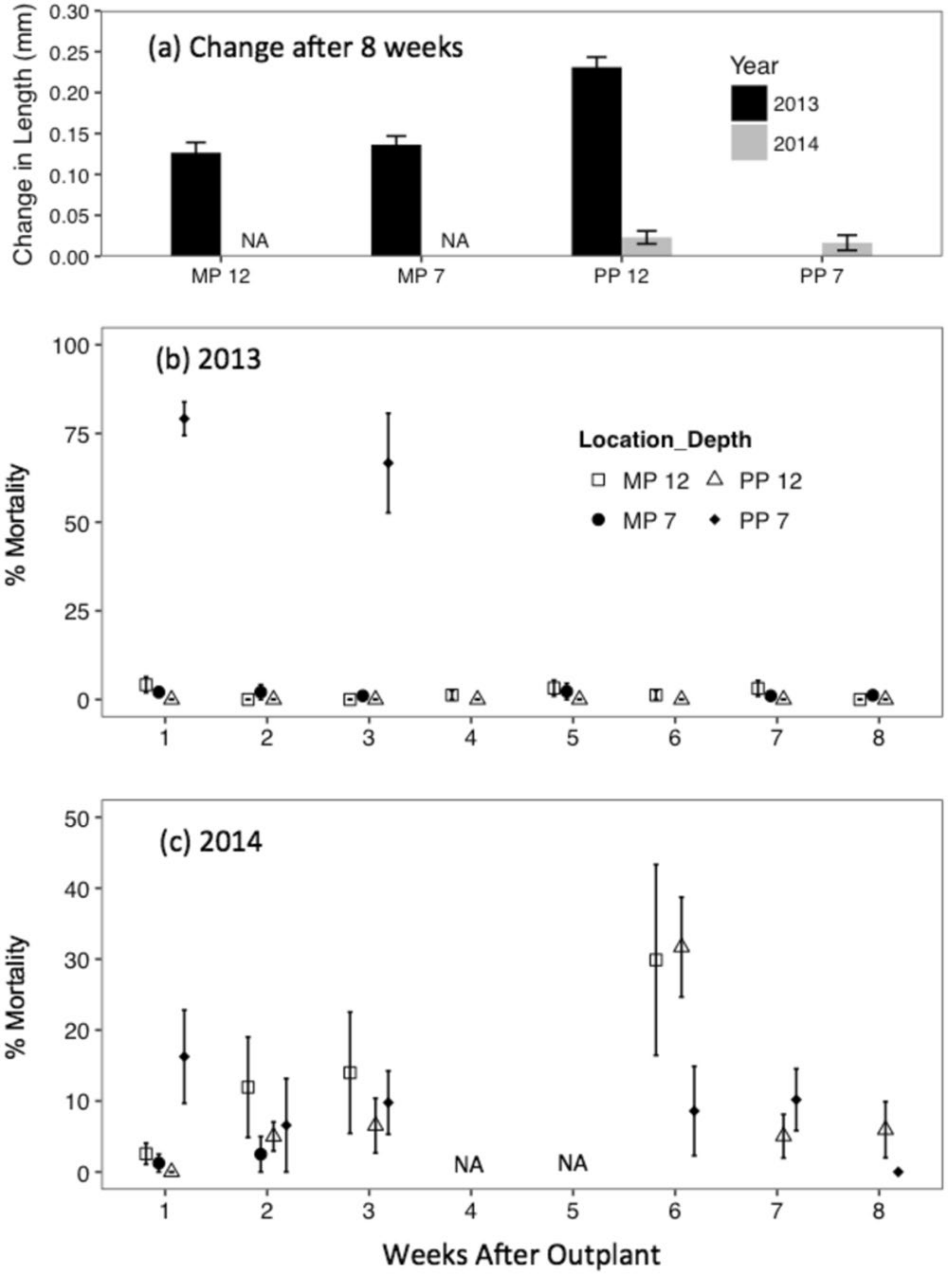
Shell growth and percent mortality of juvenile abalone in 2013 and 2014. **(a)** Mean change in shell length at the end of ~8 weeks in millimeters (mm). Black and grey bars indicate 2013 and 2014 outplants respectively. PP 7 data are not available (NA) because of high mortality at this location in 2013, and MP 12 and MP 7 data are not available (NA) because of loss of cages during an extreme storm in 2014. (**b,c)** Mean percent of dead abalone by week. Note, there is a different y-axis scale for panel (c). **(b)** 2013 mean percent mortality of Isla Natividad juvenile abalone by week. **(c)** 2014 mean percent mortality of Isla Natividad juvenile abalone by week. For panel (c), NA = mortality data not available due to weather conditions preventing divers from site visits. For all panels, MP = Morro Prieto; PP = Punta Prieta; 12 = depth of outplants in meters; 7 = depth of outplants in meters; error bars represent ± standard error.

We also conducted controlled temperature ramp experiments on juvenile abalone in the laboratory to inform interpretation of our field experiments. In these experiments, we used a thermo-coupled seawater temperature control system^30^. Although dissolved oxygen was not monitored with this system, ambient air bubbling via aquarium air pump and air stones were included in the experimental tanks to keep the seawater saturated with respect to dissolved oxygen as described in^30^. In two experimental tanks, juvenile abalone from the Isla Natividad aquaculture facility were exposed to conditions mimicking multiple 5 °C temperature ramp events for 24- and 48-hour periods. These temperature changes were measured during the 2013 field experiment, coinciding with high abalone mortality at one of the field sites (Figs 1–3). We exposed a group of juvenile abalone to ambient temperature conditions for comparison (see Fig. S5a,b for temperature series of ambient and treatment). Twenty-four animals were assigned to each group (treatment and ambient), and n = 12 of these were sampled in the treatment and ambient groups after each ramp. The second temperature ramp (Ramp 2a) of the 2013 temperature experiment was repeated with Isla Natividad juvenile abalone in 2014 (Ramp 2b). In this temperature ramp experiment, as in the previous trial, ten animals were exposed to a 5 °C ramp from 22.89 °C to 27.5 °C while 10 animals were maintained at 23.2 ± 0.2 °C. The high temperature exposure also reflected the ‘warm blob’ conditions of 2014 (27–28 °C). After 30-hr exposure, all abalone were evaluated for hemocyte viability as an indicator of sub-lethal stress.

To assess sub-lethal stress after temperature ramp exposure to the juvenile abalone, we used 1 ml sterile syringes to immediately extract a sample of hemolymph, the ‘blood’ of abalone, from the middle of each abalone foot (*n* = 10–12 abalone from each treatment group). Cells with structurally compromised membranes are ‘leaky’ and thus any hemocytes damaged by temperature stress are likely to take up tracer dyes—e.g., Trypan Blue—whereas healthy intact cells would prevent the dye from diffusing into the cytoplasm. This method of assessing non-viable and viable hemocytes has previously been used to assess sub-lethal stress in other invertebrates^31^. After extraction and placement of 1 ml of hemolymph in a 2 ml centrifuge tube, 10 ul of anti-coagulant (Alsever’s Solution, Sigma Aldrich, USA) was added to the hemolymph and the cell solution aspirated using a 100 ul pipetman. Cell viability of all the samples was then evaluated by the addition of 50 ul of Trypan Blue to each sample followed by loading a 10 ul sub-sample of the cell suspension on a hemocytometer, and manually counting both the cells that were dark blue (non-viable) and clear (viable) as viewed through a 10x magnification on a compound microscope (Lieder MC-100, American Scientific, USA). To standardize the number of cells to be counted, we sub-sampled four of the counting squares on the hemocytomer that gave a reasonable estimate of the total number of cells per volume of cell suspension. For each abalone hemolymph samples, we counted >200 total cells on the hemocytometer. Additionally, we determined whether juvenile abalone behavior was affected by the temperature ramps by placing individuals in a plastic petri dish for 1 minute, prior to hemolymph sampling, and recording how many animals moved within that time frame versus how many did not exhibit any activity.

### Statistical analysis

To analyze abalone growth and mortality data, we used the Linear mixed-effects model (LMM) and the Generalized linear mixed model (GLMM, binomial family link) with a random error component—i.e., initial size of abalone and Vexar cages nested in steel wire cages respectively—to determine if these random factors had a significant effect on the model assessment of growth and mortality. For growth, we evaluated the mean change in shell length relative to the Year and Location and Depth as fixed factors and as an initial investigation into possible differential response in biological performance. However, our main goal was to determine if changes in temperature and dissolved oxygen (or their interaction) had a differential effect on growth and survivorship. Thus, the individual change in shell length was also used to evaluate growth as a function of temperature and dissolved oxygen exposure (TI and OI) experienced over the total experimental period. For this analysis, both TI and OI were defined as a continuous factor and compared as independent factors and as interactions—i.e., TI × OI. Changes in the shell length were weighted by the number of surviving abalone in each compartment at the end of 8-week deployment. To assess abalone mortality, we evaluated the weekly proportional mortality relative to the sum of the TI and OI experienced up to each weekly census. The weekly proportion of dead versus live abalone were weighted by the total number of remaining abalone in the previous week’s census. Both TI and OI were defined as independent and continuous factors—i.e., TI × OI. We visually assessed all model fits by examining the residuals versus fitted values and did not see any over dispersion (Fig. S3). To test the significance of the comparisons in the LME models, we used analysis of variance with the Kenward-Roger approximation for degrees of freedom. To test whether the proportion of non-viable hemocytes sampled from the juvenile abalone exposed to temperature ramps was significantly different from the juvenile abalone exposed to ambient temperature conditions, we used GLMM with the sampling point as a random factor. The non-viable hemocyte data were weighted by the total number of cells counted. Multiple comparisons of mean non-viable hemocytes among treatment groups were evaluated using the Tukey post hoc test. Differences in the proportion of total inactive animals between temperature treatment and ambient groups were assessed, at each sampling point, using Chi-square tests. All analysis was done in RStudio (RStudio Inc., 2016) using the packages “lme4”, “glmm”, ‘lmerTest’, and “MASS”.

### Data availability

Data supporting the findings of this study are available from the authors and are maintained on Dropbox. Data will be made publicly available via BCO-DMO following the two-year embargo period associated with the project ends.

## Results

### Ocean Conditions

Large-scale climate variability (e.g. upwelling, EÑSO) manifested differentially between depths and on the two different sides of Isla Natividad. Local processes mitigated the larger scale variability by providing refuges to extreme environmental conditions. More specifically, the two sites separated by ~2.5 km around Isla Natividad in Baja California experienced very different exposure regimes to temperature and dissolved oxygen (Fig. 2b–e). Between 2013 and 2014, large-scale ocean conditions shifted from a relatively normal year (2013) to a weak, warm EÑSO phase (mostly due to the North Pacific warm blob which caused a warming more consistent with an extreme EÑSO event of more than 5 °C above average^32^; Fig. S2). This shift in the regional climate caused the waters on both sides of the island to warm up (Fig. 2b,d). The increase in temperatures occurred even though wind-driven upwelling was similar in both years as estimated from the cumulative wind stress for this region (2013: −19.8 Nm^−2^ d; 2014: −19.7 Nm^−2^ d).

During the 2013 experiment, ocean conditions at Morro Prieto largely followed patterns associated with wind-driven upwelling with little diurnal and tidal variability (Fig. 2b,c). In contrast, Punta Prieta conditions exhibited patterns associated with upwelling, but also maintained high variability associated with internal tides (Fig. 2b,c). While both sites experienced extreme warming during 2014 (Fig. 2d), Punta Prieta maintained high daily and tidal temperature and oxygen variability during both years. Increased warming and relatively similar overall upwelling also led to different dissolved oxygen dynamics between 2013 and 2014. In 2013, stratification was lower, waters remained well ventilated, and no low oxygen events were observed at any of the experimental locations (Fig. 2c). In 2014, warmer surface waters combined with upwelling led to increased stratification and the occurrence of several low oxygen events at both sites (Fig. 2e). Despite these similarities between sites across years, internal tidal motions at Punta Prieta led to shorter exposure to low oxygen where sites were intermittently exposed to oxygenated surface waters when low oxygen was present at depth. These records illustrate surprisingly variable conditions at scales of 100 s to 1000 s of meters that persist in the presence of strong global forcing.

At the local scale, temperature and dissolved oxygen (DO) concentrations varied greatly between years, among sites, and through time (Fig. 2 and Table 1). Exposure to extreme temperature and oxygen conditions ultimately varied between all sites and over years based on temperature and oxygen indices (Table 1). Temperatures during the field experiments were overall lower in 2013 versus 2014 (Table 1). In 2014, temperatures gradually increased during the month of August, and remained high (ave. = 23.77 +/− 1.33 °C, max = 26.04 °C) through late August and September (Fig. 2d). In both years, temperatures were up to 2 °C higher on the Vizcaino Bay side compared to the open Pacific side of the island during the first 2 weeks of the experiments (Fig. 2b,d). This was particularly evident at the shallow site (PP7m) in 2013, where, coinciding with juvenile mass mortality, seawater temperatures remained continuously above 20 °C for up to 23 hours, as opposed to only 2–3 hours at the deeper site (PP12m). Dissolved oxygen levels were high throughout the 2013 experiment (Fig. 2c and Table 1). Hypoxic conditions, which may result in sub-lethal or lethal effects on benthic marine organisms (DO < 4.6 and 2 mg/l, respectively)^32^, were not observed at the field sites during the 2013 experiment. In contrast, low DO conditions occurred at all sites in 2014 (Fig. 2e, Table 1). DO concentrations lower than 4.6 mg/l persisted for over one week at the cold-deep site in July, and concentrations as low as 1.5–2 mg/l were observed at both the shallow and deep sites for short periods of time (2–4 hours).

**Table 1:** Summary statistics of oceanographic time series (including integrated exposure).

	**Temperature**			**Dissolved Oxygen**		
	Mean (°C)	Max (°C)	Integrated (°C day)	Mean (mg/l)	Min (mg/l)	Integrated (mg/l day)
**2013**						
Morro Prieto 7	17.82	20.51	−184	6.71	5.67	−11.2
Morro Prieto 12	17.03	20.05	−249	6.73	5.36	−8.7
Punta Prieta 7	19.64	22.81	−105	7.31	6.22	−59
Punta Prieta 12	18.76	22.52	−32	6.97	5.11	−33
**2014**						
Morro Prieto 7	21.30	26.54	122	6.51	2.7	10.8
Morro Prieto 12	20.29	26.41	10.4	5.49	1.74	118
Punta Prieta 7	22.17	25.89	220	6.46	3.9	13.4
Punta Prieta 12	21.00	25.81	95	6.43	0.82	17.3
Aquaculture Tank	22.68	26.78	278	6.60	5.56	−1.4

For integrated exposure, positive values indicate exposure to extreme events.

### Abalone in the Wild: Growth and Mortality

**Figure 4:**
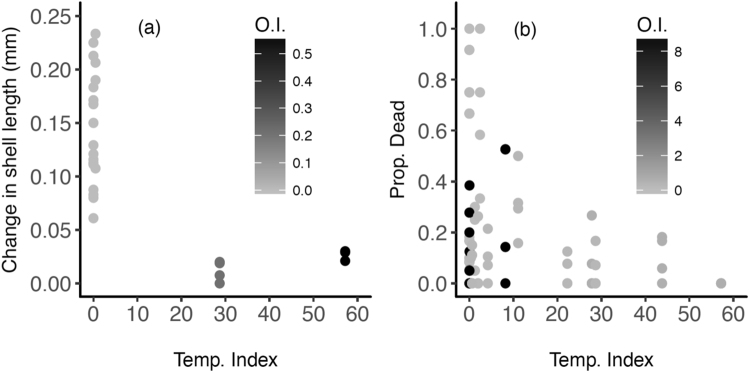
Juvenile abalone performance under oceanographic variability. **(a)** Absolute change in shell length as a function of the temperature index (degree days above 20 °C). The monochromatic gradient represents the oxygen index (days below 4.6 mg/l dissolved oxygen) to which the abalone were exposed at the same time as the temperature. **(b)** Proportional mortality of juvenile abalone as a function of the temperature index (degree days above 20 °C). The monochromatic gradient represents the oxygen index (days below 4.6 mg/l dissolved oxygen) to which the abalone were exposed at the same time as the temperature. See supplemental material for detailed temperature and oxygen index estimation. Note: The OI scales for the growth versus mortality data is scaled for the total exposure experienced at the time of sampling point—i.e., over 8 weeks versus weekly for the two datasets respectively.

**Figure 5:**
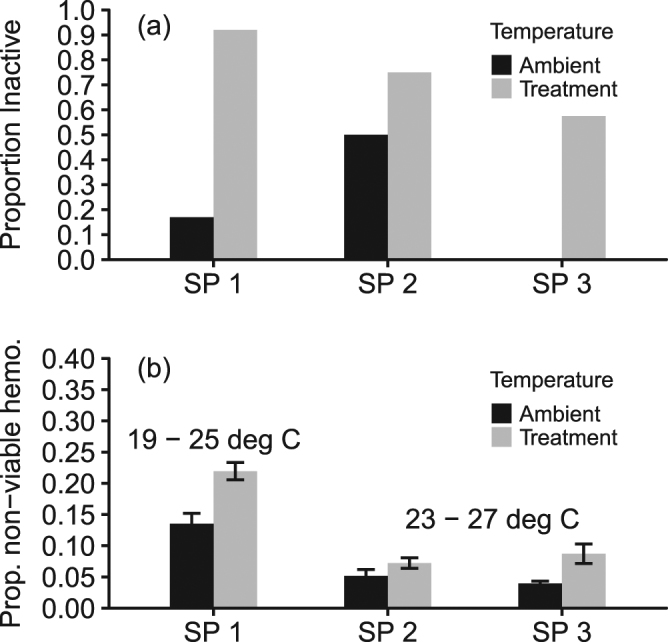
Behavioral and physiological performance of juvenile abalone. **(a)** Proportion of the total juvenile abalone inactive after elevated temperature and ambient treatments at each sampling point (SP). **(b)** Mean proportion of non-viable hemocytes after 24-h (approximately 19–25 degree C ramp, Ramp 1 followed by SP 1) and 48-h (approximately 23–27 degree C ramp, Ramp 2a followed by SP2). Mean proportion of non-viable hemocytes after 24-h in a repeated temperature ramp (Ramp 2b followed by SP 3) from 23 to 27 degrees C. Grey bars indicate cell response to temperature ramp treatment and black bars indicate cell viability response to ambient temperature conditions. For all hemolymph samples, n > 200 total number of cells were counted on a hemocytometer. Error bars are ± standard error; SP = sampling point.

Juvenile green abalone performance was highly variable between the two years and among locations (Fig. 3). Compared to the changes in growth for the juvenile abalone outplanted at Morro Prieto 12 m in 2013, the shell growth was significantly less in 2014 (Fig. 3a; *F* = 57.20; *p* < 0.001) and among locations (*F* = 6.79; *p* < 0.001; Fig. 3a; Table S1). The largest growth was observed for the abalone outplanted at the deep, warm site (PP12) in 2013 (*F* = 6.79; *p* < 0.001; Fig. 3a, Table 2). Initial size had a negligible effect on the absolute change in shell length *(F* = 1.81*; p* > 0.05*;* Table 2A). The results indicate that high temperature exposure (TI) had a positive effect on the slope of the growth response (*F* = 20.93, *p* < 0.001, Fig. 4a, Table 2A); however, hypoxic exposure (OI) appeared to have a more significant negative effect on the slope of the growth response (*F* = 22.25; *p* < 0.001; Fig. 4a, Table 2A). Results also indicate a significant interactive effect of TI and OI on the slope of growth response (*F* = 23.41; *p* < 0.001; Fig. 4a, Table 2A). Juvenile abalone held in the laboratory throughout the 2014 experiment experienced increased growth despite exposure to temperature increases from 20 °C to ~27 °C that was similar to the natural variation at Punta Prieta (Fig. 5a; Fig. S4a). At the end of the experiment, the laboratory abalone had approximately ten-fold more positive growth than the abalone outplanted at any of the field sites in 2014 (Fig. S4a). Although laboratory abalone were exposed to high temperatures, these animals did not experience low oxygen conditions (Fig. S4b).

**Table 2:** Model statistics for growth and mortality response to oceanographic variability.

Model		Estimate	SE	DF	*p*-value
(**A**) Growth	(Intercept)	0.37	0.15	28	*
*y* = Initial Size + TI x OI + ~(1|Initial Size) + *e*	Initial Size	−0.15	0.10	28	0.14
	TI	0.16	0.05	28	**
	OI	−28.42	9.29	28	**
	TI x OI	0.20	0.07	28	**
	**Random Effect**	**(Intercept)**	**Residual**		
	Std. Dev.	0.04	0.02		
**Model**		**Estimate**	**SE**	***z*** **-value**	***p*** **-value**
(**B**) Mortality	(Intercept)	−3.22	0.77	−4.19	***
Y = TI * OI + (1|Steel cage/Vexar cage) + *e*	TI	−0.04	0.01	−4.87	***
	OI	0.20	0.05	4.33	***
	TI x OI	0.02	0.01	4.36	***
**Random Effects**					
**Groups:**	**Variance**	**Std. Dev**			
Compartment	2.32	1.52			
Cage	3.96	1.99			
Number of observations	258				
Vexar cages	32				
Steel cages	8				

(**A**) Linear mixed-effects model (LMM) evaluation of growth in response to initial size, temperature (TI) and oxygen indices (OI). For this model, the initial sizes of the juvenile abalone at time of outplant were considered a random factor. *y* = absolute change in shell length in millimeters; *e* = error term. (**B**) Generalized linear mixed model (GLMM) evaluation of mortality response to temperature (TI) and oxygen indices (OI). For this model, Vexar cages nested in steel wire cages were considered as a random factor. *y* = proportion dead; *e* = error term. For both tables: *p < 0.05; **p < 0.01; ***p < 0.001 significance.

Mortality varied among sites and through time (Fig. 3). Mortality during the first 3 weeks of deployment was highest among sites in 2013, with near 100% mortality at the Punta Prieta shallow site but not at the other locations (Fig. 3b; Table S1). After this initial localized mass mortality event, mortality was low at the remaining sites throughout the experiment (between 0–3% weekly; Fig. 3b). Mortality was overall greater but steadier throughout the experiment in 2014 compared to 2013, at all sites (between 0–30% weekly; Fig. 3c). After 8 weeks, 76% (±16.32%) of the juveniles survived in 2013 at the sites unaffected by mass mortality, but only 20.94% (±6.63%) survived in 2014. Proportional mortality had a small but significant negative response to increases in exposure to high temperature (Fig. 4b; Table 2B). In contrast, proportional mortality had a large and positive response to increased exposure to low DO (Table 2B). However, the GLMM analysis indicates a significant interactive effect between TI and OI where temperature imposed a mild recovery on the effects of low oxygen alone (*p* < 0.001; Table 2B). Mortality was virtually non-existent in the laboratory samples (n = 2 out of 80 abalone over 8 weeks) in 2014 despite high mortality rates observed in the field sites.

### Abalone in the Lab: Physiology and Behavioral Responses

In the laboratory temperature ramp experiments, the proportion of inactive juvenile abalone after exposure to elevated temperature treatments were consistently higher than the proportion of inactive juvenile abalone under ambient temperature conditions (Fig. 5a). At the first sampling point (Fig. 5a, SP 1), 91.7% versus 16.7% of the total juvenile abalone were inactive in treatment versus ambient temperature conditions (*χ*^2^ = 15.6, *p* = 0.0002). At the second sampling point (Fig. 5a, SP 2), activity levels did not differ between treatments (75% inactive) and ambient groups (50% inactive; *χ*^2^ = 1.6, *p* = 0.21). At the third sampling point (Fig. 5a, SP 3), a greater proportion of animals were inactive in the treatment (60%) compared to the ambient group (0%; *χ*^2^ = 8.6, *p* = 0.003).

After exposure to a rapid change in temperature from 19 to 25 °C over 4 hours (Fig. S5a), the first samples (Fig. 5b, SP 1) of cell viability taken from the juvenile abalone indicated that the proportion of non-viable hemocytes were significantly higher relative to the proportion of non-viable hemocytes as measured from juvenile abalone exposed to ambient temperature conditions (Table S2, p < 0.001). After an additional 24-hour exposure to a secondary temperature ramp to ~27 °C, a second measure (Fig. 5b, SP 2) of cell viability indicated that the proportion of non-viable hemocytes remained significantly higher under elevated temperatures versus ambient conditions (Table S2, p < 0.001). A similar ramp in temperature treatments from 23 to 27.5 °C (Fig. S5b,c, Ramp 2b, SP 3) also resulted in significantly higher proportion of non-viable hemocytes in the juvenile abalone versus those abalone exposed to ambient conditions (Table S2, p < 0.001).

Overall, the higher temperature treatment groups at each sampling point consistently responded with significantly higher proportion of inactive individuals and non-viable hemocytes. Additionally, the behavioral and physiology data also indicate an overall reduction of both the proportion of inactive individuals and of non-viable hemocytes over time independent of the temperature treatment. These results indicate that juveniles can physiologically acclimate to short-term increases in temperature relatively quickly, and along with 98% (n = 2/80 animals) survivorship in the aquaculture control tanks, these data indicate that the extreme warming that occurred across the whole region in 2014 did not have direct negative impacts on abalone mortality.

## Discussion

Elevated temperatures associated with EÑSO events or loss of kelps, the primary food of abalones, have been suggested as the leading cause of growth depression in both juvenile and adult red abalone (*Haliotis rufescens*)^33^. However, our results concur with other controlled experiments which suggest that oxidative stress or hypoxia rather than temperature has a larger negative effect on growth^19,34^. At younger and smaller post-larval and early recruit stages, juvenile abalone (e.g., *H. rufescens*) not only grow less under oxidative stress, but mortality can also result after long exposures. While we did not perform oxidative stress experiments, our results support evidence that hypoxic events are more likely than anomalously high temperatures to cause depressed growth in juvenile abalone in natural systems.

Although physical factors are sub-lethally and lethally impacting juvenile abalone performance, our results also indicate that some juvenile abalone can survive both EÑSO-like high temperatures and hypoxic events. Juvenile abalone can acclimate to temperature and even metabolically adjust after 1 month combined exposure to temperature and dissolved oxygen stress^20,35^. Our study indicates that although juvenile abalone exhibit evidence of physiological stress when exposed to elevated temperatures over short periods (hours to two days), the data also indicate that juvenile abalone can rapidly acclimate as indicated by the reduction in both the inactivity of movement and non-viable hemocytes independent of temperature treatments. Thus, the ability of juvenile abalone to acclimate to elevated temperatures may be critical in the overall biological performance when considered over longer tidal and seasonal cycles. While acclimation to elevated temperatures may explain the high survivorship and positive growth observed in the laboratory abalone over two months, similar responses to low oxygen exposure are not known. Our field study indicates that the two stressors do have interactive effects on abalone performance, and individual adaptation will also likely be affected. Improving our understanding of multiple stressors and the range of physiological thresholds coupled to genetic diversity will be an important step for guiding management actions and understanding marine reserve function^36,37^. Without question, the EÑSO is a major climatic driver of ecosystem function and fishery productivity in eastern Pacific coastal systems^32^. However, additional work is needed to more accurately predict the differential response of organisms at multiple life stages to the effects of dynamic changes in temperature, hypoxia, and ocean acidification.

We recognize that we did not include the effects of pH, salinity and other biologically relevant variables for this investigation of multiple stressors on juvenile abalone, although pH in this region is known to co-vary with oxygen and temperature. Ocean acidification or changes in pH have been reported to have negative impacts on abalone larvae^38^, and the growth and mortality of newly settled juveniles^34^. Although our results suggest that the combination of temperature and oxygen (or pH) has higher effects on growth and mortality than individual stressors, future experiments will need to include a more comprehensive set of covariates to understand the suite of oceanographic impacts on multiple biological processes.

Punta Prieta is a designated marine reserve that allowed us to get an initial understanding of how physical variables would influence juvenile abalone performance if the local fishing community implemented a restocking program in a marine reserve. The mortality differences at the shallow versus deeper site only ~200 meters away effectively demonstrated the need for further understanding of local scale dynamics in predicting climate change impacts. Despite the conditions at the shallow site being favorable, as indicated by both traditional knowledge and the lack of hypoxic events in this area, other local conditions are clearly driving the survivorship of juvenile abalone at this shallow site. In contrast to the shallow site, the deeper site within the Punta Prieta reserve had the highest survivorship in both years. An alternative explanation to direct physical forcing as the causative agent may be that some sites are driven by the physical conditions that favor disease or algal blooms that are harmful to abalone^15,16,39–41^. For example, abalone mortality was greater at shallower sites during a 2011 mass mortality event near Mendocino, CA where genomic evidence indicated a toxic algal bloom as the causative agent^13^. Although we have not presented evidence for such indirect effects, our results highlight the importance of understanding how large-scale forcing manifest at the local spatial scales and the key factors (e.g. low dissolved oxygen exposure along with other possible covariates) that may be negatively influencing recruitment.

Mass mortalities in marine systems have been widespread resulting in significant decline of affected species over entire regions^10,11^, but mortality can be locally patchy because of resistant individuals and subpopulations or local scale oceanographic variability^12,26,42^. Individuals from either resistant subpopulations or climate refuges could ultimately affect the persistence of species both in the near- and far-future scenarios if these adaptive subpopulations persist long enough to reproduce and pass on their plasticity. Thus, we must identify and protect the diversity of sub-populations in areas of stable recruitment in order to prepare for continued climate change.

## Electronic supplementary material

Supplemental online material
