# Supplementary material for: Local oceanographic variability influences the performance of juvenile abalone under climate change
Source: Sci Rep. 2018 Apr 3;8:5501. doi: 10.1038/s41598-018-23746-z (PMC5882807; doi:10.1038/s41598-018-23746-z)

**Supplementary Information for: “Local oceanographic variability influences the performance of juvenile abalone under climate change”**

C. A. Boch^1,2,*^, F. Micheli^1^, M. AlNajjar^3^, S. G. Monismith^3^, J. M. Beers^1^, J. C. Bonilla^4^, A. M. Espinoza^5^, L. Vazquez-Vera^6^, and C. B. Woodson^7^

^1^Hopkins Marine Station, Stanford University, Pacific Grove, CA 93950, USA

^2^Monterey Bay Aquarium Research Institute, Moss Landing, CA 95039, USA

^3^Dept. of Civil and Environmental Engineering, Stanford University, Stanford, CA 94305, USA

^4^Sociedad Cooperativa de Producción Pesquera La Purisima, Bahia Tortugas, Baja California Sur, México

^5^Sociedad Cooperativa de Producción Pesquera Buzos y Pescadores, Isla Natividad, Baja California Sur, México

^6^Comunidad y Biodiversidad A.C., Calle Isla del Peruano No.215, Guaymas, Sonora, 85448, México

^7^College of Engineering, University of Georgia, Athens, GA 30602, USA

*Corresponding author:* Charles A. Boch; Monterey Bay Aquarium Research Institute, 7700 Sandholdt Road, Moss Landing, CA 95039; email: cboch@mbari.org; phone: 831-775-1849; fax: 831-775-1620


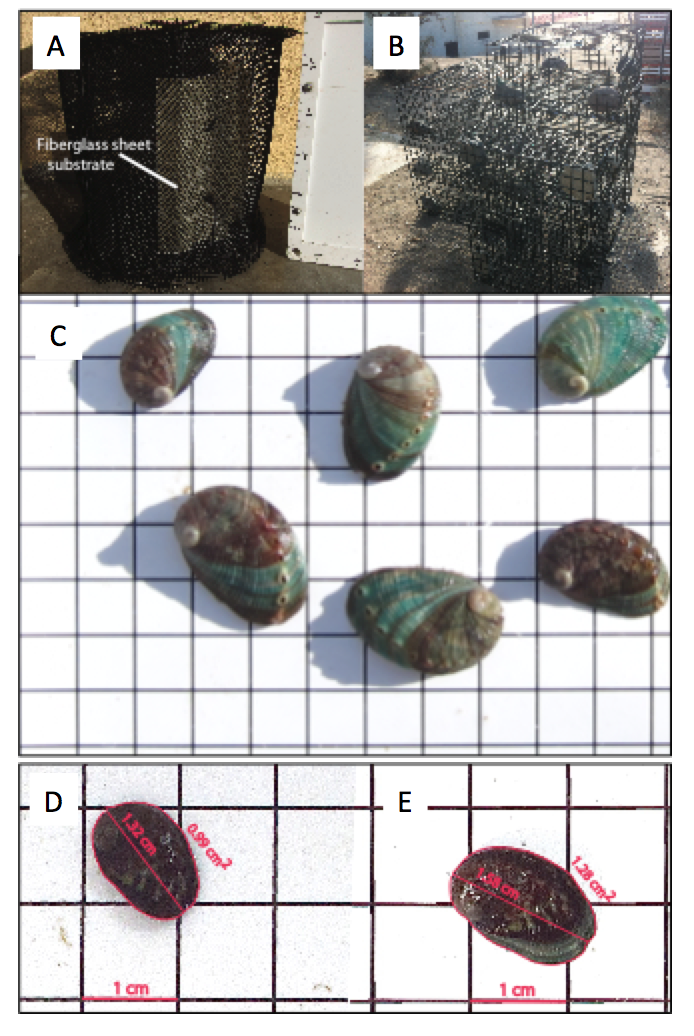


**Supplementary Figure S1.** *Haliotis fulgens* juvenile abalone outplant modules and estimating size. **A.** Vexar mesh cages (8 mm mesh size) with fiberglass substrates. **B.** Steel wire cages (5x5 cm mesh size; 85x70x30 cm in size; approximately 20 kg each) with the four rocks in the corners of the cages. Additionally, steel wire cage modules were tied down with extra weights of about 50 kilograms (underwater weight) at the experimental site locations. **C.** Sample juvenile abalone micrograph on waterproof paper grid (16x16 cm, 1 cm increments). **D.** Size_initial_ measurement of juvenile abalone shell—i.e., 1 day before outplant—using ImageJ software (https://imagej.nih.gov/ij/). **E.** Size_final_ measurement of the same juvenile abalone from panel D—i.e., size at final day of experiment (~ 8 weeks). To determine individual growth, each individual abalone was visually matched with each surviving abalone using the unique color and banding patterns on the surface of the shell. All abalone were dabbed dry with paper tissue prior to photographing with a Canon S10 Powershot (Canon USA Inc.) mounted on a tripod.


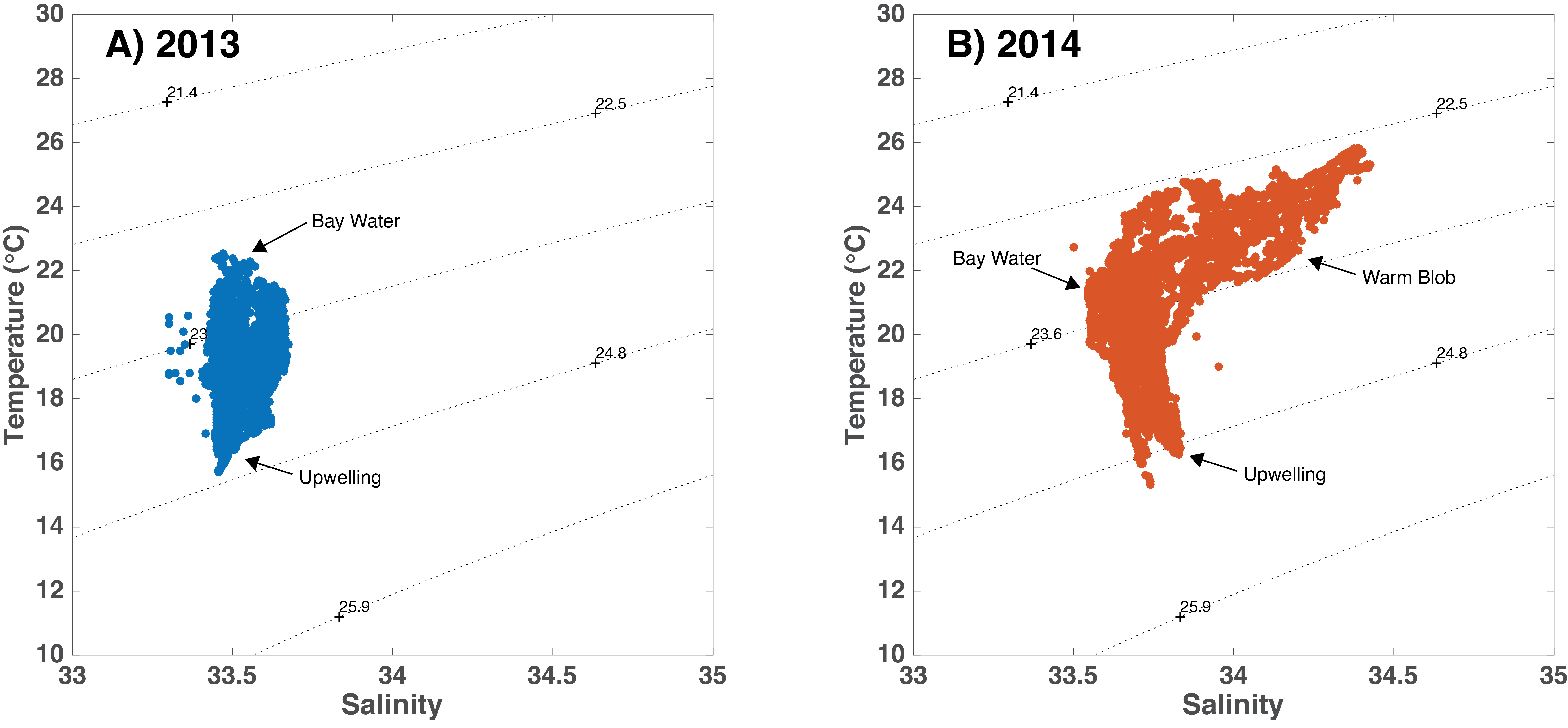


**Supplementary Figure S2.** Temperature-Salinity diagrams from Punta Prieta for both years during experiment. **A.** 2013 showing relatively constant salinity, and general locations of upwelled and Vizcaino Bay water. **B.** 2014 showing addition of warm, salty Pacific Warm Blob water that was advected into region during the experiment. Morro Prieto shows similar results without the presence of Vizcaino Bay water (not shown because of a lost CTD in 2014).


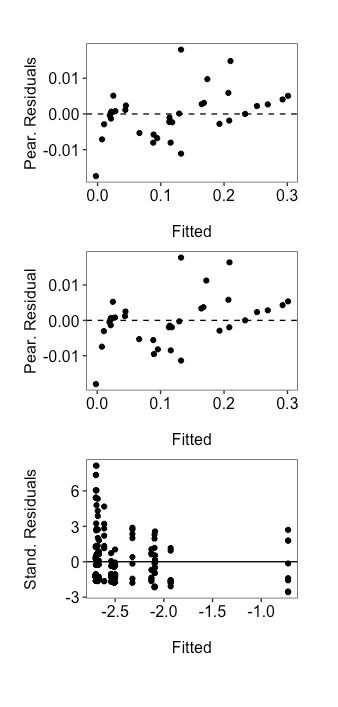


**Supplementary Figure S3.** Dispersion of residuals versus model fits. **A.** Pearson residuals versus LMM fitted values for the model y (Change in Shell Length) = Year + Location Depth + (1 | Initial Size) + *e.* Initial size is input as a random factor. Data are weighted by the number of juvenile abalone in each compartment at the end of 8-week deployment. **B.** Pearson residuals versus LMM fitted values for the model y (Change in Shell Length) = TI x OI + (1 | InitialS) + *e*. Initial size is input as a random factor. Data are weighted by the number of juvenile abalone in each compartment at the end of 8-week deployment. **C.** Standardized residuals versus GLM fitted values for the model y (Proportional Mortality) = TI x OI + *e*. D. Data are weighted by the number of juvenile abalone remaining for each week after weekly SCUBA dive checks for dead abalone—i.e., empty shells.


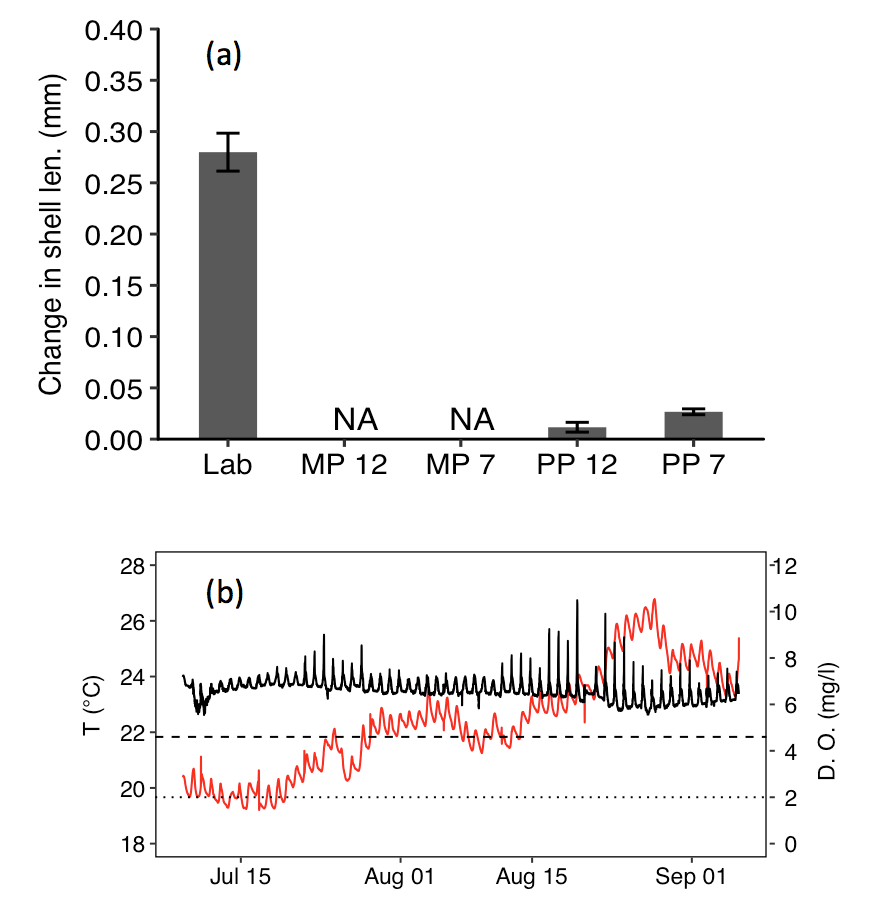


**Supplementary Figure S4.** **(a)** Absolute change in shell length for juvenile abalone *in situ* and in the Is. Natividad laboratory after 8 weeks, in 2014. NA = not available due to storms removing the outplants. **(b)** Temperature and dissolved oxygen time series of Isla Natividad (Baja California, Mexico) laboratory tank during the experiment in 2014.


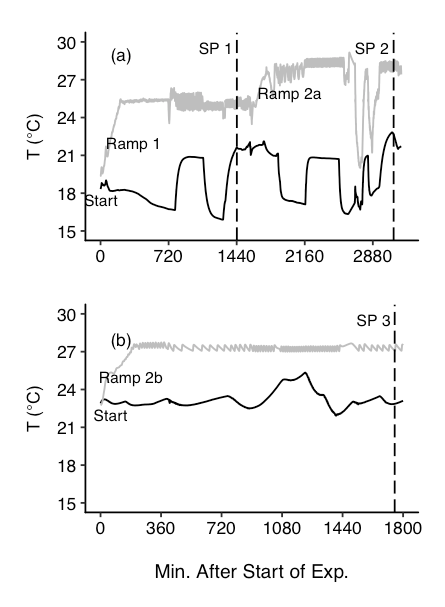


**Supplementary Figure S5.** **a)** Time series of temperature ramp (grey line) and ambient (black line) exposures for Isla Natividad juvenile abalone. **b)** Time series of high temperature treatment (grey line) and ambient (black line) temperature exposure to Isla Natividad juvenile abalone. For both a,b the *x*-axis represents minutes after start of experiment and SP = sampling points of hemolymph.

**Supplementary Table 1.** Analysis of abalone growth response by year, outplant location, and depth. Comparative evaluation of the change in shell length with the 2013 MP 12 data as the comparative reference (Linear mixed-effects model). For this model, the initial sizes of the juvenile abalone at time of outplant were considered a random factor. *y* = absolute change in shell length in millimeters; *e* = error term; * = *p* < 0.05; ** = *p* < 0.01; *** = *p* < 0.001 significance.

| Changes in Shell Length |  | **Estimate** | **SE** | **DF** | ***p*-value** |  |
| --- | --- | --- | --- | --- | --- | --- |
| *y* = Year + Location Depth + ~(1\|Initial Size) + *e* | (Intercept) | 0.14 | 0.02 | 28 | *** |  |
|  | Year (2014) | -0.21 | 0.03 | 28 | *** |  |
|  | Location Depth (MP7) | -0.01 | 0.03 | 28 | 0.70 |  |
|  | Location Depth (PP12) | 0.09 | 0.02 | 28 | *** |  |
|  | Location Depth (PP7) | 0.09 | 0.04 | 28 | * |  |
|  |  |  |  |  |  |  |
|  | **Random Effects** | **Variance** | **Std. Dev.** |  |  |  |
|  | Initial Size | 0.002 | 0.04 |  |  |  |
|  | Residual | 0.002 | 0.04 |  |  |  |
|  |  |  |  |  |  |  |
| Analysis of Variance Table of Type III with Kenward-Roger approximation for degrees of freedom | | | | | | |
|  | Sum sq. | Mean Sq. | NumDF | DenDF | F-value | *p*-value |
| Year | 0.103 | 0.103 | 1 | 72.7 | 57.2 | *** |
| Location_Depth | 0.037 | 0.012 | 3 | 67.6 | 6.79 | *** |
|  |  |  |  |  |  |  |
|  |  |  |  |  |  |  |
|  |  |  |  |  |  |  |
|  |  |  |  |  |  |  |
|  |  |  |  |  |  |  |
|  |  |  |  |  |  |  |
|  |  |  |  |  |  |  |

**Supplementary Table 2.** GLMM comparison of cell viability sampled from abalone exposed to temperature treatments and Tukey post hoc multiple comparison of means. For this model, the ambient data at SP 1 was considered as the reference group to initially determine in what direction the abalone responded. *y* = proportion of cells non-viable; *e* = error term; * = *p* < 0.05; ** = *p* < 0.01; *** = *p* < 0.001 significance.


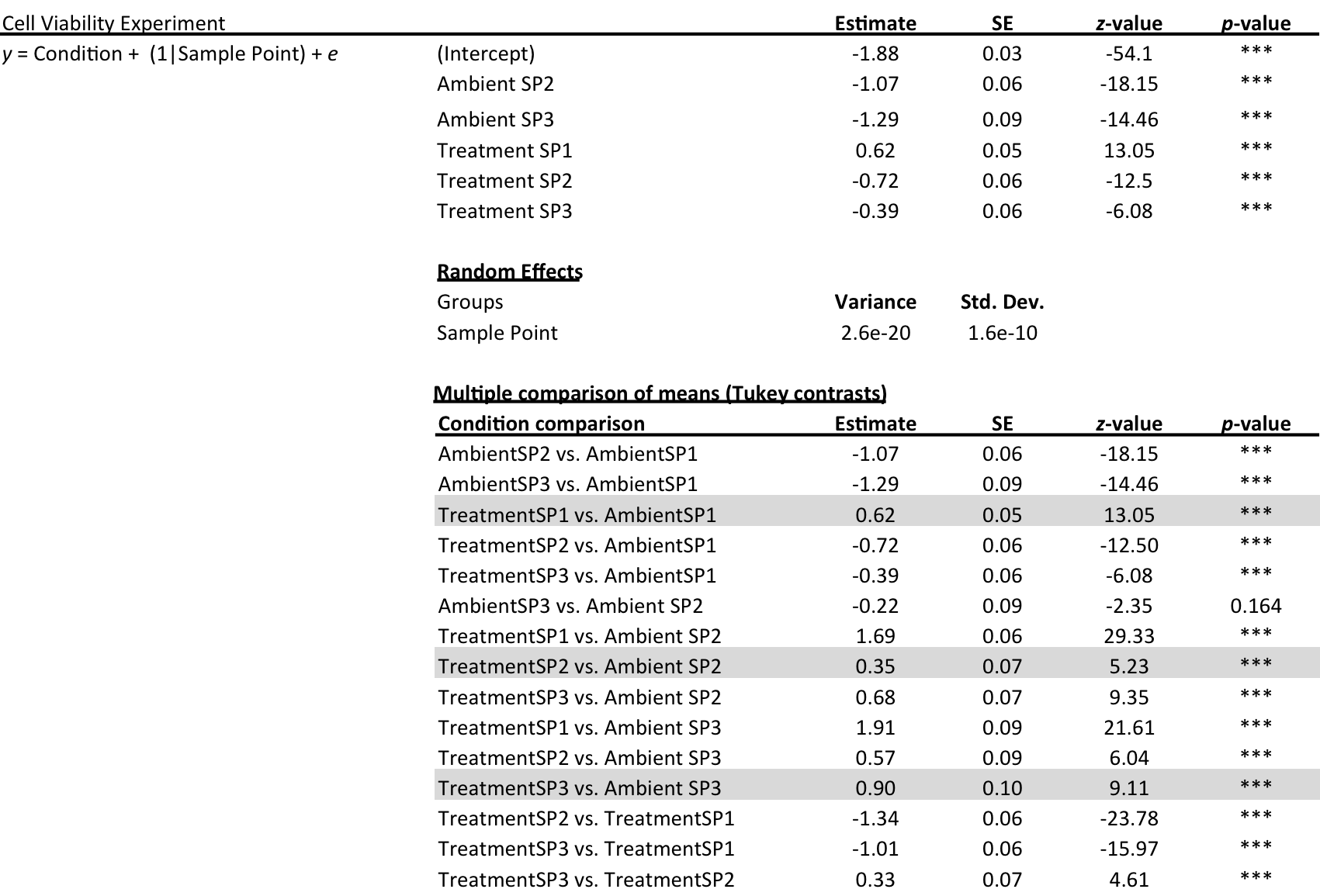

Supplement: Supplementary file 1 — Supplemental online material [file 41598_2018_23746_MOESM1_ESM.docx]
